# Factors affecting resolution of subretinal fluid after selective retina therapy for central serous chorioretinopathy

**DOI:** 10.1038/s41598-021-88372-8

**Published:** 2021-04-26

**Authors:** Akika Kyo, Manabu Yamamoto, Kumiko Hirayama, Takeya Kohno, Dirk Theisen-Kunde, Ralf Brinkmann, Yoko Miura, Shigeru Honda

**Affiliations:** 1 Department of Ophthalmology and Visual Science, Osaka City University Graduate School of Medicine, Osaka, 545-8585 Japan; 2 Medical Laser Center Lübeck, Lübeck, Germany; 3 Institute of Biomedical Optics, University of Lübeck, Lübeck, Germany; 4 Department of Ophthalmology, University of Lübeck, Lübeck, Germany

**Keywords:** Medical research, Risk factors, Retinal diseases

## Abstract

The purpose of this study was to investigate the factors of clinical outcome of selective retina therapy (SRT) for central serous chorioretinopathy (CSC). This retrospective study included 77 eyes of 77 patients, who were treated with SRT for CSC and observed at least 6 months after the treatment. SRT laser (527 nm, 1.7 µs, 100 Hz) was used for treatment. The mean best-corrected visual acuity (logMAR), central macular thickness (CMT) and central choroidal thickness were changed from baseline to at 6-months follow-up with significant difference. The multivariate analyses found that the rate of change (reduction) in CMT was associated with focal leakage type on fluorescein angiography (FA) (*p* = 0.03, coefficient 15.26, 95% confidence interval 1.72–28.79) and larger baseline CMT (*p* < 0.01, coefficient − 0.13, 95% confidence interval − 0.13 to − 0.05). Complete resolution of subretinal fluid was associated with nonsmoking history (*p* = 0.03, odds ratio 0.276, 95% confidence interval 0.086–0.887) and focal leakage type on FA (*p* < 0.01, odds ratio 0.136, 95% confidence interval 0.042–0.437). These results may be useful for predicting the therapeutic effectiveness of SRT.

## Introduction

Central serous chorioretinopathy (CSC) is known as a retinal disorder causing subretinal fluid (SRF) and resulting visual disturbances including metamorphopsia, central scotoma, reduced visual acuity and loss of contrast sensitivity. Most cases of acute CSC are self-limited and resolved spontaneously, but on the other hand, it may be persistent and treatment-resistant. After recurrent or prolonged SRF, some patients experience permanent visual impairment with retinal pigment epithelium (RPE) atrophy^1–4^. Several treatment methods were suggested for patients with prolonged SRF. Recently, because conventional laser photocoagulation may lead to irreversible scotoma in the central macula^5,6^, half-dose photodynamic therapy (PDT) and subthreshold microsecond-pulsed laser (SMPL) treatment were selected for persistent SRF with CSC^7–11^.

Selective retina therapy (SRT) was developed as a novel and unique laser procedure in which the RPE is selectively broken down through a microbubble formation within RPE cells^12–14^. This treatment does not induce thermal diffusion in surrounding tissues, which enables selective RPE disruption without damaging the neural retina or choroid. Several reports have revealed that SRT was effective for CSC, diabetic macular edema (DME), persistent subretinal fluid after retinal detachment surgery, etc^15–23^.

Previously, we reported the safety of SRT for CSC of Japanese patients using microperimetry after three months^18^. We also revealed the predictive factors associated with retinal thickness after SRT treatment for DME^21^. In this study, we carried out a retrospective investigation to evaluate the factors which may affect the therapeutic effectiveness of SRT on CSC after the treatment.

## Methods

### Subjects

This study was approved by the Ethical Committee of Osaka City University Graduate School of Medicine (No. 2009 and 2421), carried out on the basis of the Declaration of Helsinki, and SRT was registered with University hospital Medical Information Network (UMIN) (No. 000005396 and 000010471). Written informed consent was obtained from all patients prior to enrolment. This study investigated 77 eyes of 77 CSC patients (61 eyes of 61 males and 16 eyes of 16 females), who underwent SRT in the Department of Ophthalmology at Osaka City University Hospital between June 2011 and December 2016 and were followed-up for at least 6 months. The mean age of patients was 50.5 years (range, 29–78 years). Table 1 shows baseline characteristics of the patients in this study.

**Table 1:** Patient characteristics at baseline.

**Characteristics**	
Number of patients	77 cases (77 eyes)
Sex	Male 61, female 16
Age; mean (range)	50.5 (29–78)
Hypertension (%)	12 (15.6)
Smoking (%)	35 (45.5)
**Duration of symptom (months) (%)**	
≤ 6	22 (28.6)
6 < and ≤ 12	14 (18.2)
12 < and ≤ 24	17 (22.1)
24 < or unknown	24 (31.2)
**Number of episodes (%)**	
First	41 (53.2)
Second or more	36 (46.8)
**Leakage type on FA**	
Focal	42 (54.5)
Diffuse	35 (45.5)
BCVA (logMAR); mean (range)	0.08 (1.70 to − 0.18)
CMT: mean µm (range)	316 (121–511)
CCT: mean µm (range)	341 (117–574)

*FA*: fluorescein angiography; *BCVA*: best corrected visual acuity; *logMAR*: logarithm of the minimum angle of resolution; *CMT*: central macular thickness; *CCT*: central choroidal thickness.

### Clinical observations and SRT enrolment

The clinical examination to diagnose CSC included measurement of the best-corrected visual acuity (BCVA), slit-lamp microscopy with a contact lens or noncontact lens, funduscopy, fundus autofluorescence, fluorescein and indocyanine green angiography (FA and IA), and optic coherence tomography (OCT) (SPECTRALIS; Heidelberg Engineering GmbH, Heidelberg, Germany). When the following inclusion/exclusion criteria were fulfilled, observation, conventional laser photocoagulation (if leakage was not within the sub- to parafoveal lesion), half-dose PDT, and SRT were presented as treatment options, and SRT was performed, if patients desired. All patients underwent the ophthalmic observations at baseline and at monthly follow-up after the treatment (FA was performed before and every 3 months after SRT).

### SRT inclusion and exclusion criteria

Inclusion criteria for selection of patient treated with SRT were as follows:

1. minimum age of 20 years,
2. subjective symptoms of central scotoma, metamorphopsia, or decline of visual acuity,
3. history of more than 3 months with no sign of improvement of CSC diagnosed with OCT,
4. presence of SRF on macular region on OCT,
5. presence of active leakage on FA, and
6. presence of choroidal vascular hyperpermeability on IA.

Ophthalmologic exclusion criteria were as follows:

1. macular diseases with SRF caused by the disease other than CSC,
2. history of other laser treatments for CSC, such as conventional laser, PDT or SMPL,
3. absence of leakage in FA, and
4. presence of choroidal neovascularization on FA and IA.

Systematic exclusion criteria were as follows:

1. inflammatory disease,
2. bleeding tendency and anticoagulation therapy,
3. presence or possibility of pregnancy,
4. untreated hypertension and diabetes mellitus, and
5. taking of diuretic, such as acetazolamide or spironolactone.

### SRT method

The SRT system (Medical Laser Center Lübeck, Lübeck, Germany) utilizes a Q-switched frequency-doubled neodymium-doped yttrium lithium fluoride (Nd:YLF) laser, frequency doubled to a wavelength of 527 nm. In a single irradiation, a short 1.7 µs laser pulse is repeated 30 times at a repetition rate of 100Hz. The laser beam was adjusted such that the irradiation diameter on the retina was approximately 200 µm with a top hat beam profile under the use of a 1.05× magnification Mainster central field contact lens.

All SRT laser irradiations have been conducted by one ophthalmologist only. The treatment procedure of SRT is principally based on the previous study by Roider et al.^20^, and briefly as follows; test irradiations were conducted outside of the pathological central region, mostly near the vascular arcade. Beginning with the lowest energy (about 50–60 μJ), the irradiation energy was increased stepwise (every 10–20 μJ), where about 4–10 different energies were tested twice each until a weakly visible spot is funduscopically observed. Followed by the test laser irradiations, FA was conducted. In order to achieve RPE cell disruption with minimally required energy, the energy, with which weakly positive leakage was detected in FA, was chosen as the initiation energy for the treatment.

**Figure 1:**
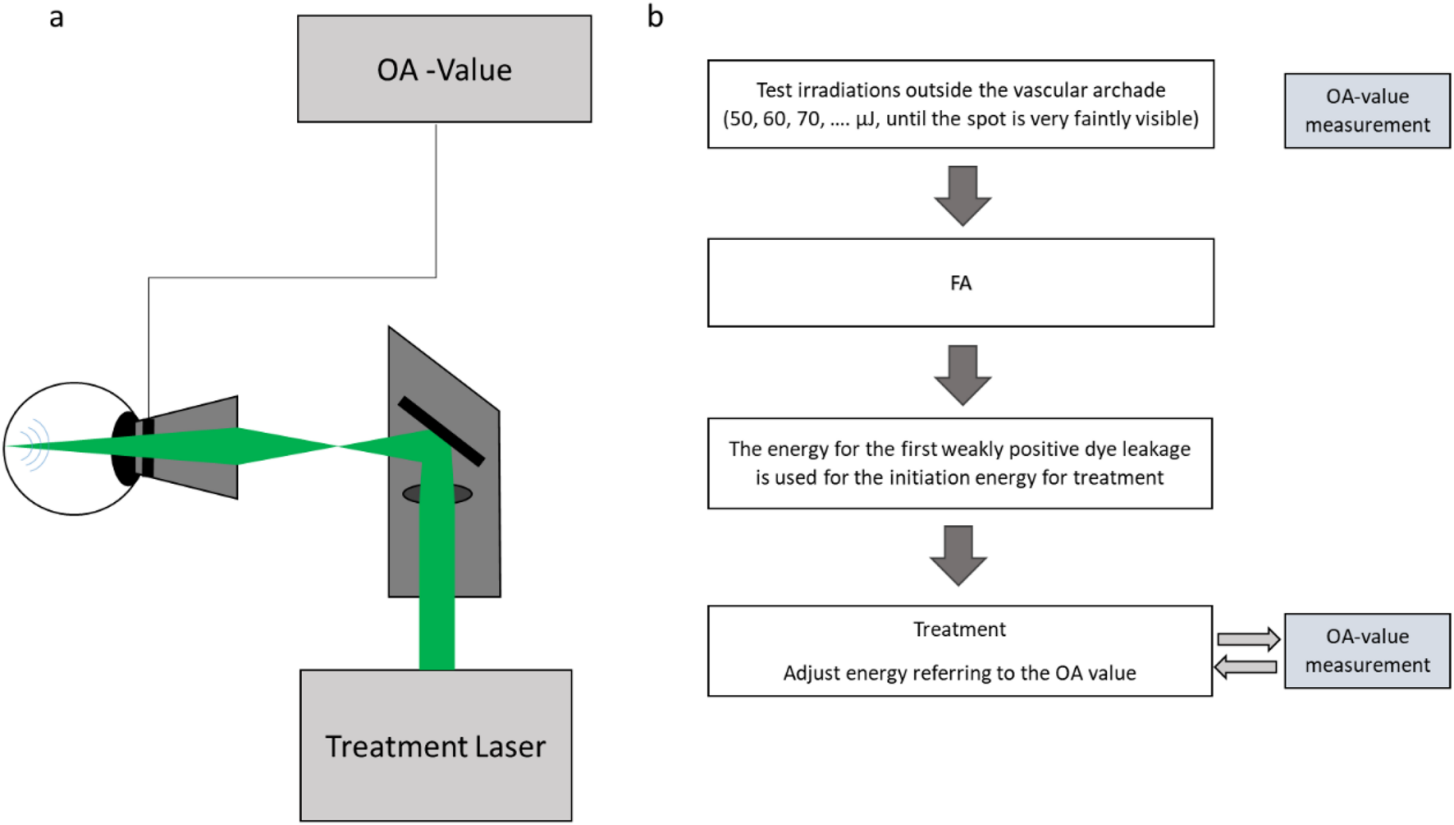
Schematic diagram of (**a**) the setup and (**b**) the treatment procedure of selective retina therapy (SRT).

Additionally, a real-time optoacoustic dosing control was used. It detects the pressure waves emitted after microbubble formation within the RPE, which are responsible for the selective RPE disruption. The pressure waves were measured with an ultrasonic transducer embedded in the Mainster contact lens (Medical Laser Center Lübeck, Lübeck, Germany) and processed by a software calculating an optoacoustic value (OA-value) after each exposure. The OA value was found to correlate with angiographic leakage. A detailed description of the optoacoustic dosimetry was published by Schuele^24^. According to our previous study, an OA value of OA = 70 indicates 50% probability of leakage on FA (Effective Dose (ED) 50), and OA=112 indicates 90% probability (ED90) in the used system^18^. After deciding the treatment energy, the treatment was performed at and around the leakage point assessed with FA, giving an interval between spots of about one spot diameter. The treatment proceeded while ensuring the OA value at least around the ED90 or higher, indicating RPE disruption, with a minimally required energy. Figure 1 shows the setup and the treatment workflow of the SRT.

### Outcome measures

BCVA, OCT, and FA were performed before treatment and 1, 3 and 6 months after SRT. Central macular thickness (CMT), and central choroidal thickness (CCT) were also investigated. With regard to BCVA, changes of ≥ 0.2 in logMAR unit were considered significant. A change in CMT ≥ 15% compared with the pre-treatment baseline was regarded as significant as previously described^21^. Since there is no previous report on the significance criteria for the rate of change in CCT, 15% was set to be significant based on the rate of change in CMT. SRT was considered effective if CMT decreased significantly compared to baseline, and as ineffective if this was not the case. In cases with no previous history of any treatment in the fellow eye, changes in CCT between the treated and fellow eyes were compared at 1, 3 and 6 months after SRT. As factors that might influence the rate of change in CMT and resolution of SRF 6 months after SRT, we evaluated sex, age, previous hypertension, smoking history (current or former smoking longer than 5 years, independent of the amount), duration of symptom (months), number of disease episodes (first and second or more), history of medicine, leakage type on FA (focal or diffuse), baseline BCVA, baseline CMT and baseline CCT. Previous hypertension, smoking history, duration of symptom, number of episodes were obtained using the questionnaire at initial treatment period.

### Statistical analysis

Changes in BCVA (logMAR), CMT and CCT from baseline were assessed using a paired t-test with Bonferroni correction. Correlation between change in CCT with treated and fellow eyes were assessed using a paired t-test with Bonferroni correction. In order to assess the associations between the changes of CMT after SRT treatment and clinical factors among SRT treated patients, we performed a univariable linear regression analyses with the change value of CMT at 6 months as the function of each clinical characteristic. The correlation between resolution of SRF and clinical factors was also assessed by using univariable logistic regression analysis. Factors showing *p* values < 0.2 in univariate analyses were used for a multivariate analysis. IBM SPSS Statistics 24.0 (IBM Japan, Ltd., Tokyo, Japan) was used for statistical analysis, in which *p* values < 0.05 were regarded as significant.

## Results

**Figure 2:**
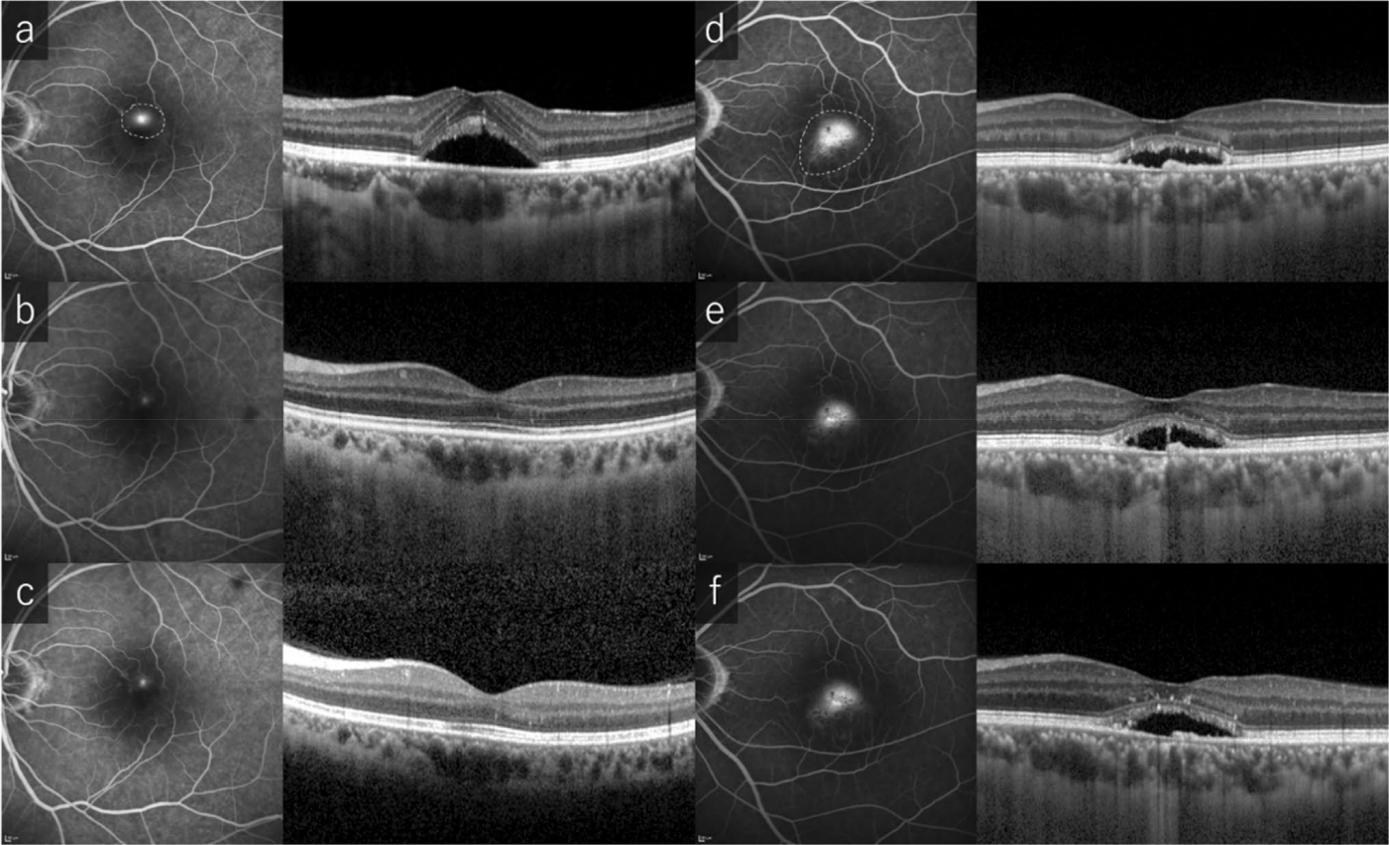
Fluorescein angiography and horizontal line of optic coherence tomography images of patients at baseline (**a**, **d**), 3 months follow-up (**b**, **e**) and 6 months follow-up (**c**, **f**) after selective retina therapy (SRT). Extent of SRT irradiation (within yellow dotted line). (**a**)–(**c**): 47-year-old, non-smoker male with an 11-year history of vision loss in the left eye. The number of SRT irradiation was 8 and laser energy was ranged from 81 to 103 µJ (optoacoustic, OA value was ranged from 61 to 1009). Focal leakage was disappeared and complete resolution of subretinal fluid (SRF) was seen at 3- and 6-months follow-up. Central macular thickness (CMT) was decreased from 412 to 222 µm. (**d**)–(**f**): 51-year-old, smoker male with a 5-month history of vision loss and abnormal visual field in the left eye. The number of SRT irradiation was 10 and laser energy was ranged from 82 to 101 µJ (OA value was ranged from 98 to 476). Diffuse leakage and SRF was remained at 6 months follow-up. CMT was slightly decreased from 281 to 275 µm.

**Figure 3:**
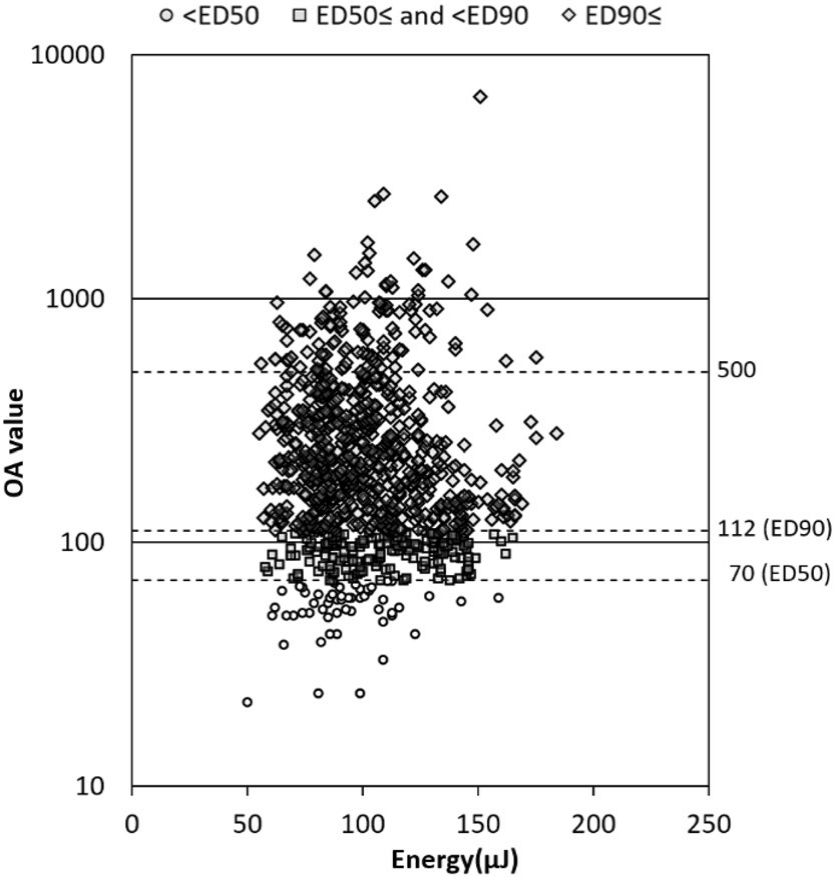
SRT irradiation energy and optoacoustic (OA) values. The scatter plot shows the correspondence between irradiation energy and optoacoustic value at each irradiation spot in all cases.

Typical two cases of CSC treated with SRT are shown in Fig. 2. The mean number of irradiations in one SRT was 12.0 ± 7.4 (range, 1–42). Per patient, the mean number of irradiations with < ED50 (OA < 70) was 0.7 ± 1.3 (4.9% ± 8.7%), the mean number of irradiations with ≥ ED50 but <ED90 (70 ≤ OA < 112) was 2.2 ± 3.1 (13.9% ± 16.0%), and the mean number of irradiations with ≥ ED90 (OA ≥ 112) was 9.0 ± 6.1 (81.1% ± 19.4%) (Fig. 3).

### Clinical and morphological changes

**Figure 4:**
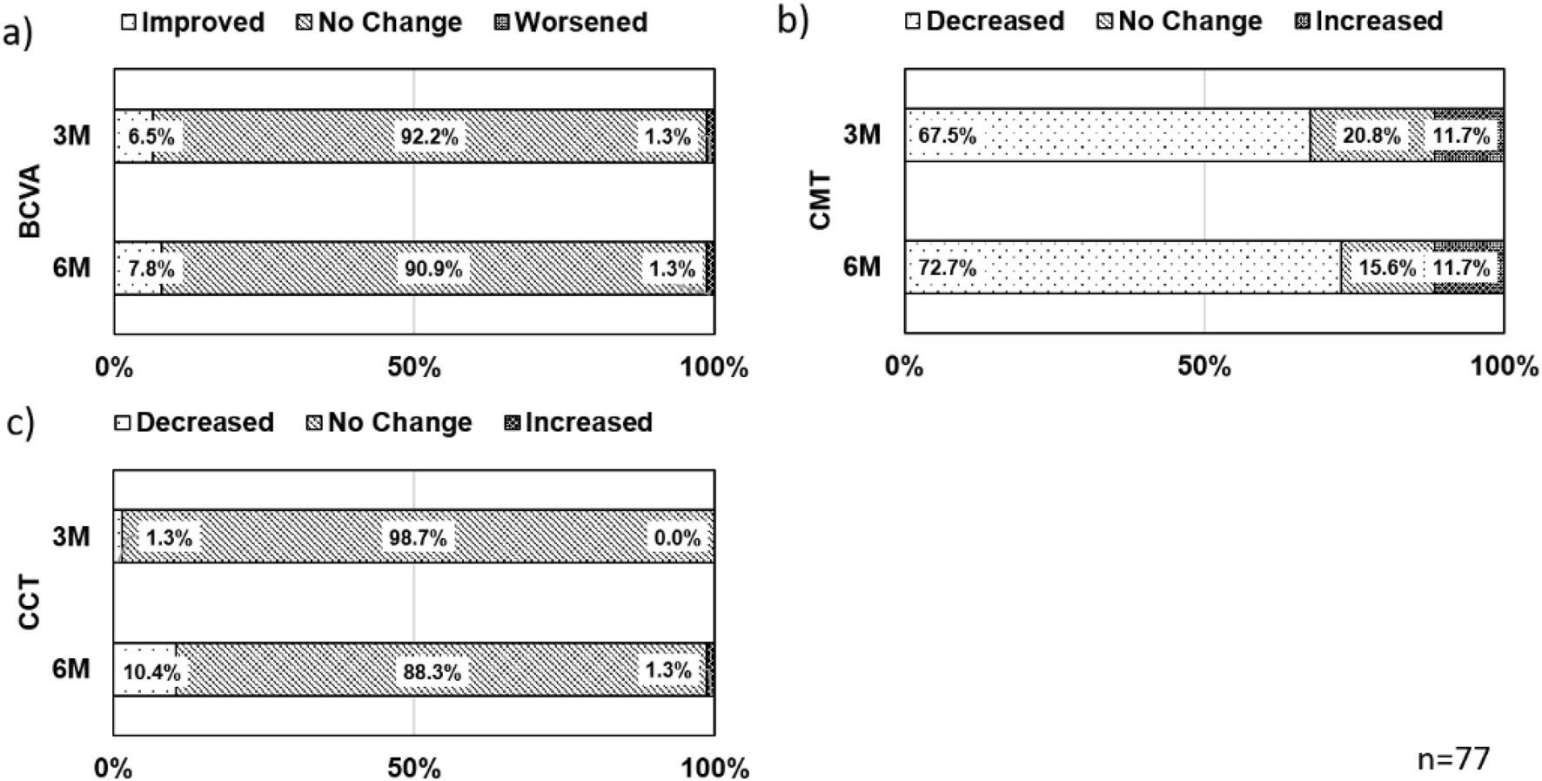
Time course of individual changes in best corrected visual acuity (BCVA), central macular thickness (CMT), and central choroidal thickness (CCT). (**a**) Proportions of patients whose BCVA was improved, unchanged, or worsened by ≥ 0.2 from baseline at 3 and 6 months after treatment. The proportion of patients with improved BCVA was 6.9% at 3 months and 7.8% at 6 months. (**b**) Proportion of patients whose CMT reduced, unchanged, or increased by ≥ 15% from baseline at 3 and 6 months after treatment. The number of patients with reduced CMT increased (from 67.5 to 72.7%) from 3 to 6 M. (**c**) Proportion of patients whose CCT reduced, unchanged, or increased by ≥ 15% from baseline at 3 M and 6 M after treatment. The number of patients with reduced CCT increased (from 1.3 to 10.4%) from 3 to 6 M.

Changes of visual acuity and OCT findings during follow-up are shown in Table 2. Mean BCVA (logMAR) was 0.08 ± 0.28 before SRT, 0.07 ± 0.31 after 1 month, 0.04 ± 0.31 after 3 months, and 0.04 ± 0.29 after 6 months, with significant difference after 3 and 6 months (1 month, *p* = 1.00; 3 months, *p* < 0.01; 6 months, *p* < 0.01). Individually, after 3 months BCVA had improved in 6.5% of patients, was unchanged in 92.2% and had worsened in 1.3%, and after 6 months had improved in 7.8%, was unchanged in 90.9%, and had worsened in 1.3% (Fig. 4a).

**Table 2:** Changes of visual acuity and optical coherence tomography findings during follow-up.

	Baseline	1 M	*p* value	3 M	*p* value	6 M	*p* value
BCVA (logMAR); mean ± SD	0.08 ± 0.28	0.07 ± 0.31	1.00	0.04 ± 0.31	< 0.01	0.04 ± 0.29	< 0.01
CMT (µm); mean ± SD	316 ± 89	246 ± 85	< 0.01	246 ± 94	< 0.01	218 ± 82	< 0.01
CCT (µm); mean ± SD	352 ± 97	349 ± 98	0.34	339 ± 94	< 0.01	330 ± 95	< 0.01
Complete resolution of SRF, N (%)	–		–	33 (42.9)	–	46 (59.7)	–

Mean CMT was 316 ± 89 μm before SRT, 246 ± 85 μm after 1 month, 246 ± 94 μm after 3 months, and 218 ± 82 μm after 6 months, showing a significant decrease after 1, 3 and 6 months (1 month, *p* < 0.01; 3 months, *p* < 0.01; 6 months, *p* < 0.01). Individually, after 3 months CMT had decreased in 67.5% of patients, was unchanged in 20.8%, and had increased in 11.7%, and after 6 months had decreased in 72.7%, was unchanged in 15.6%, and had increased in 11.7% (Fig. 4b). Complete resolution of SRF was observed in 33 eyes (42.9%) 3 months after SRT and in 46 eyes (59.7%) 6 months after SRT.

Mean CCT was 352 ± 97 μm before SRT, 349 ± 98 μm after 1 month, 339 ± 94 μm after 3 months, and 330 ± 95 μm after 6 months, showing a significant decrease after 3 and 6 months compared to the baseline (1 month, *p* = 0.34; 3 months, *p* < 0.01; 6 months, *p* < 0.01). Individually, after 3 months CCT had significantly decreased (more than 15%) in 7.8% of patients, was unchanged in 90.9%, and had increased in 1.3%, and after 6 months had decreased in 14.3%, was unchanged in 77.9%, and had increased in 7.8% (Fig. 4c).

**Figure 5:**
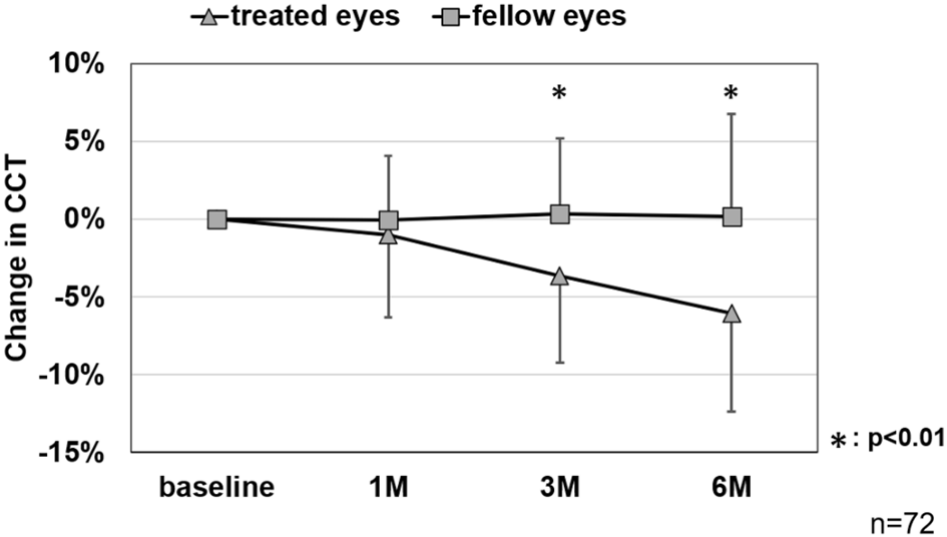
Changes in central choroidal thickness (CCT) between the treated and fellow eye mean CCT at baseline was 351 ± 97 μm in treated eyes and 312 ± 92 μm in fellow eyes with a significant difference (*p* < 0.05). Mean change in CCT was − 1.0 ± 5.3% in treated eyes and − 0.1 ± 4.2% in fellow eyes after 1 month, − 3.6 ± 5.6% and 0.4 ± 4.8% after 3 months, and − 6.0 ± 6.3% and 0.2 ± 6.6% after 6 months, showing a significant difference after 3 and 6 months (1 month, *p* = 0.73; 3 months, *p* < 0.01; 6 months, *p* < 0.01).

There were 5 cases with previous treatment history in fellow eye, we thus examined the difference between treated and fellow eye in 72 cases. Among these 72 cases, mean CCT at baseline was 351 ± 97 μm in treated eyes and 312 ± 92 μm in fellow eyes with a significant difference (*p* < 0.05). Mean change in CCT was − 1.0 ± 5.3% in treated eyes and − 0.1 ± 4.2% in fellow eyes after 1 month, − 3.6 ± 5.6% and 0.4 ± 4.8% after 3 months, and − 6.0 ± 6.3% and 0.2 ± 6.6% after 6 months, showing a significant difference after 3 and 6 months (1 month, *p* = 0.73; 3 months, *p* < 0.01; 6 months, *p* < 0.01). There was no significant change in the CCT in these fellow eyes over 6 months compared to the baseline (Fig. 5).

### Factors associated with retinal findings

**Figure 6:**
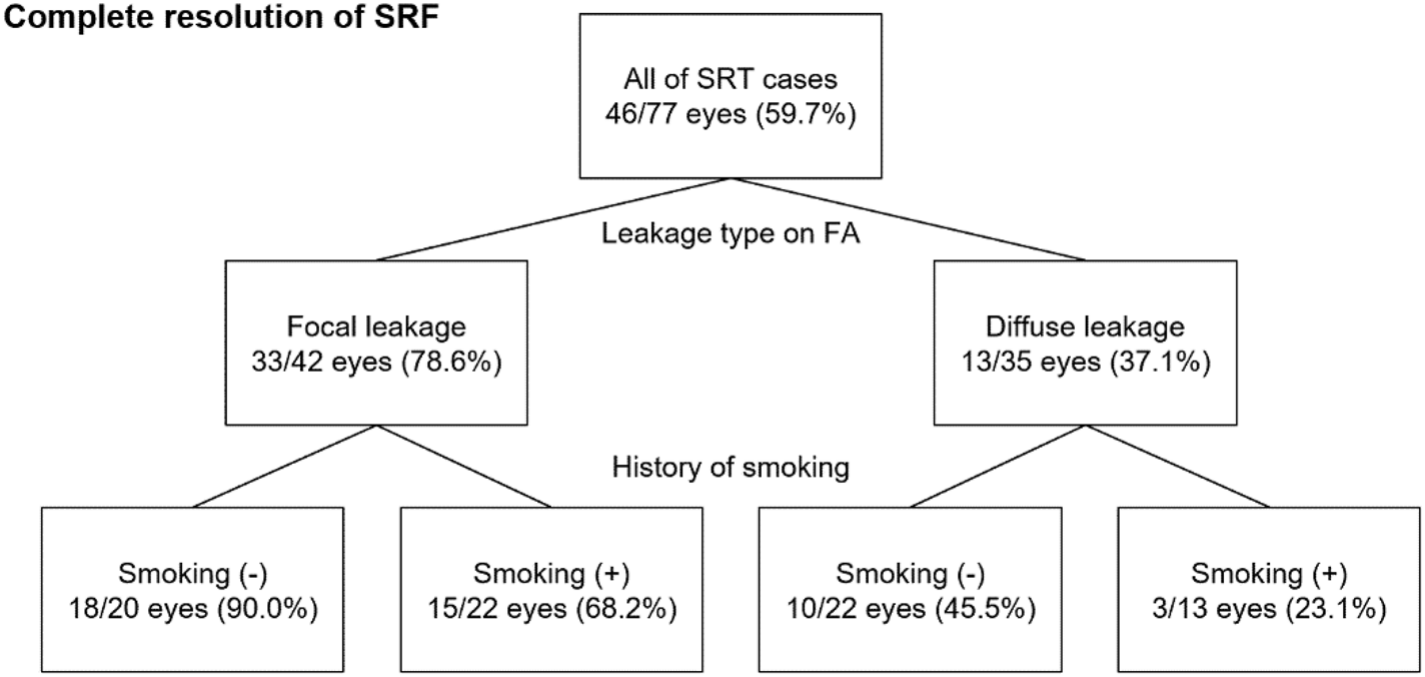
Decision tree classified by the factors associated with the complete resolution of subretinal fluid (SRF). In this study, the overall complete resolution of SRF within 6 months after SRT was 46 out of 77 cases (59.7%). For patients with focal leakage on FA (n = 42), the complete resolution of SRF was seen in 33 cases (78.6%) whereas those with diffuse leakage (n = 35) that was seen in 13 cases (37.1%). For the non-smoker patients with focal leakage (n = 20), complete resolution of SRF was seen in 18 cases (90.0%), whereas for the smoker patients (n = 22) that was seen in 15 cases (68.2%). For the non-smoker patients with diffuse leakage (n = 22), complete resolution of SRF was seen in 10 cases (45.5%), whereas for the smoker patients (n = 22) that was seen only in 3 cases (23.1%).

Univariate and multivariate regression analysis are shown in Tables 3 and 4. The multivariate linear regression analysis found that the rate of change in CMT at 6 months after SRT was significantly associated positively with focal leakage type on FA (*p* = 0.03, coefficient 15.26, 95% confidence interval 1.72–28.79) and larger baseline CMT (*p* < 0.01, coefficient − 0.13, 95% confidence interval − 0.13 to − 0.05) (Table 3). The multivariate logistic regression analysis found that the resolution of SRF at 6 months after SRT was significantly associated negatively with history of smoking (*p* = 0.03, odds ratio 0.276, 95% confidence interval 0.086–0.887) and positively with focal leakage type on FA (*p* < 0.01, odds ratio 0.136, 95% confidence interval 0.042–0.437) (Table 4). Figure 6 shows the decision tree classified by the factors associated with complete resolution of SRF used as the basis for the result of multivariate analysis.

**Table 3:** Univariate and multivariate analysis of factors associated with the rate of change in central macular thickness at 6 months after selective retina therapy.

	Univariate			Multivariate		
	Co	95% CI	*p* value	Co	95% CI	*p* value
Age	− 0.464	(− 1.149 to 0.220)	0.18	− 0.545	(− 1.172 to 0.082)	0.09
Sex (male: female)	− 3.795	(− 21.116 to 13.525)	0.66			
Hypertension	− 8.841	(− 28.133 to 10.451)	0.36			
Smoking	7.947	(− 6.065 to 21.959)	0.26			
Duration of symptom (months)	− 0.520	(− 6.379 to 5.340)	0.86			
Number of episodes (first: second or more)	2.913	(− 11.173 to 16.999)	0.68			
History of medicine	− 0.901	(− 15.177 to 13.375)	0.90			
Leakage type on FA (focal: diffuse)	20.073	(6.717–33.428)	< 0.01	15.257	(1.724–28.790)	0.03
Baseline BCVA (logMAR)	− 4.904	(− 29.808 to 19.999)	0.70			
Baseline CMT (µm)	− 0.159	(− 0.229 to 0.089)	< 0.01	− 0.125	(− 0.199 to − 0.051)	< 0.01
Baseline CCT (µm)	− 0.028	(− 0.101 to 0.045)	0.45			

*Co*: coefficient; *CI*: Confidence interval.

**Table 4:** Univariate and multivariate analysis of factors associated with complete resolution of subretinal fluid at 6 months after selective retina therapy.

	Univariate			Multivariate		
	OR	95% CI	*p* value	OR	95% CI	*p* value
Age	0.996	(0.953–1.042)	0.87			
Sex (male: female)	1.199	(0.394–3.651)	0.75			
Hypertension	1.421	(0.388–5.202)	0.60			
Smoking	0.529	(0.210–1.332)	0.177	0.276	(0.086–0.887)	0.03
Duration of symptom (months)	1.132	(0.774–1.656)	0.52			
Number of episodes (first: second or more)	1.385	(0.553–3.468)	0.49			
History of medicine	1.925	(0.745–4.976)	0.18	1.721	(0.578–5.124)	0.33
Leakage type on FA (focal: diffuse)	0.161	(0.059–0.441)	< 0.01	0.136	(0.042–0.437)	< 0.01
Baseline BCVA (logMAR)	1.633	(0.289–9.217)	0.58			
Baseline CMT (µm)	1.002	(0.997–1.007)	0.47			
Baseline CCT (µm)	1.005	1.000	0.06	1.003	(0.998–1.009)	0.26

*OR*: odds ratio; *CI*: Confidence interval.

### Adverse events

During this study, no patient developed intraocular inflammation, haemorrhage, or other event attributable to laser irradiation.

## Discussion

This is the first report to investigate the factors affecting effectiveness of SRT for CSC patients. SRT may reportedly lead to the complete resolution of SRF after 3 months in 19–74% of patients with chronic CSC^15–19^. In our current study, mean CMT in CSC patients was significantly decreased 6 months after SRT in 72.7% of patients. Complete resolution of SRF was seen in 59.7% of all cases. Although there are some differences in the inclusion criteria and the follow-up period, our current result shows the treatment effectiveness as high as in the previous reports with SRT.

Many reports have suggested various risk factors for developing CSC, such as endogenous or exogenous steroids, coagulation abnormalities, infection of Helicobacter pylori, males, pregnancy, hypertension, use of antibacterial agents, history of smoking, alcohol intake, sleep disturbance, oxidative stress, type A personality, psychological stress, etc^2,25–30^. Matet et al. reported that risk factors of recurrence were significantly associated with thick subfoveal choroidal thickness and weak leakage on FA in CSC^31^. In the current study, according to classification of FA, the focal leakage type had a higher rate of CMT reduction and a higher rate of SRF resolution than the diffuse leakage type independently of the disease duration. Focal leakage type is considered to have a smaller range of damaged RPE cell lesion than diffuse leakage. As a condition for SRF to be resolved after SRT, regrowth and activation of RPE is indispensable. It is possible that the larger the range of damaged RPE cell lesion, the less possibility for the surrounding RPE cells to regrowth and establish a better functional RPE. CMT was decreased in 72.7% of all cases, and the decrease rate of CMT was associated with baseline CMT. The SRF in chronic CSC tends to be shallower rather than dome shaped in comparison with acute CSC^32^. Due to those facts, diffuse leakage type and smaller baseline CMT of chronic CSC may reflect high treatment resistance shown in SRT.

PDT and SMPL treatment are conspicuous treatment modalities for CSC cases, with many reports that has been published with regard to their efficacy. In the PLACE Trial, half-dose PDT was superior to SMPL treatment, with 67% of PDT-treated patients achieving a complete resolution of SRF, as compared to 29% of SMPL patients at 7–8 months after treatment^7^. Ho et al. reported that 87% of half-dose PDT patients and 50% of SMPL patients had complete resolution of SRF at 3 months after treatment^8^. PDT induces choroidal vascular changes and remodeling, a decrease of choroidal thickening to near-normal levels, and an associated decrease in extravascular leakage^33^. SMPL enables the sublethal heating of RPE cells sparing iatrogenic side effects on conventional laser photocoagulation include scotomas, epiretinal fibrosis and choroidal neovascularization. Previous basic studies suggested that SMPL may lead to different protein expressions such as heat shock proteins and aquaporin^34–36^. Different from SMPL, SRT, where much shorter pulse is applied, causes microbubble formation around melanosomes, resulting in selective disruption of RPE cell without heating cells, thus without damaging surrounding tissues^12–14^. After SRT, RPE regeneration follows by migrating and proliferating of RPE cells, which cover the lesion of cell defect. Although there are differences in the mechanism of action between PDT, SMPL and SRT, all treatments are considered to be able to lead to the normalization and remodeling of RPE cell function, resulting in the absorption of SRF.

Interestingly, CCT was also significantly decreased 6 months after SRT in this study, although the individual assessment with the 15% cutoff value, defined as for CMT, showed a small number of patients with its decrease. In PDT, there are many examinations reporting a choroidal thickness decrease after treatment^7–9^. In SMPL treatment, several reports showed no significant changes in choroidal thickness before and after treatment, while others showed a slight decrease^7,8,10,11^. Park et al. reported no significant changes in choroidal thickness 3 months after SRT^19^. We cannot explain this discrepancy yet, requiring the data accumulation in the future studies. There are, however, some possible mechanisms to be discussed. Although SRT affects primarily only RPE cells, it has been shown in the previous basic studies that SRT may increase secretion of active matrix metalloproteinases 2 and pigment epithelium-derived factor and decrease vascular endothelial growth factor (VEGF) into the choroidal side after SRT irradiation^37,38^. The direct impact of these factors on the choroidal thickness has not been proven yet to date, but there are increasing clinical evidence that several cytokines including VEGF may be correlated to the choroidal thickness in different macular disorders^39,40^. Thus we assume that the change in the cytokine secretion profiles from RPE through SRT may affect choroidal thickness, although the impact is much lower than through PDT, which may reportedly reduce choroidal thickness up to 12–21%^2,9^.

One of the important findings of this study is the fact that smoking history was associated with a poor SRF resolution. Smoking is not only associated with developing CSC, but poor visual prognosis and longer treatment needed in CSC patients^41^. Cotinine, the major nicotine metabolite, reduces RPE cell repair, wound healing ability and phagocytotic activity in vitro^42^. Fujihara et al. reported that chronic cigarette smoke develops evidence of oxidative damage with ultrastructural degeneration to the RPE and Bruch's membrane, and RPE cell apoptosis^43^. Since the mechanism of response to SRT is based on the improvement of RPE cell function, smoking, which can cause RPE dysfunction may strongly influence the therapeutic effect of SRT. As shown in the Fig. 6, to investigate history of smoking and leakage type on FA may be informative for predicting visual function and anatomical effects after SRT.

In this study, we have conducted multivariate analyses for two different dependent variables, the reduction rate of CMT and the complete resolution of SRF. Although these two matters are considered to be highly correlated, we obtained slightly different results. As the reason it is assumed, first, due to the different analysis method, one is with continuous variable and one is with nominal variable. The second reason could be due to the possible existence of another factor required for the complete resolution of the SRF. In order to clarify this point, we would propose both analyses for further studies, too.

In conclusion, our study showed that effectiveness of SRT was independently associated with smoking history, leakage type on FA and baseline CMT for CSC patients over 6 months of follow-up. This study was limited by the inclusion of a small number of patients and by a non-randomized and retrospective study design. Nevertheless, the analysis was conducted by examining relatively larger subject than the previous reports. We are thus convinced that these results are useful in considering future prospective clinical studies on SRT. Further prospective studies with a larger number of patients is desired to confirm the association of these factors with the outcomes of SRT.
